# A Surprising Prevention Success: Why Did the HIV Epidemic Decline in Zimbabwe?

**DOI:** 10.1371/journal.pmed.1000414

**Published:** 2011-02-08

**Authors:** Daniel T. Halperin, Owen Mugurungi, Timothy B. Hallett, Backson Muchini, Bruce Campbell, Tapuwa Magure, Clemens Benedikt, Simon Gregson

**Affiliations:** 1 Harvard University School of Public Health, Boston, Massachusetts, United States of America; 2 Ministry for Health and Child Welfare, Harare, Zimbabwe; 3 Imperial College London, London, United Kingdom; 4 Independent consultant, Harare, Zimbabwe; 5 United Nations Population Fund, Harare, Zimbabwe; 6 Zimbabwe National AIDS Council, Harare, Zimbabwe; 7 Biomedical Research and Training Institute, Harare, Zimbabwe

## Abstract

Daniel Halperin and colleagues examine reasons for the remarkable decline in HIV in Zimbabwe, in the context of severe social, political, and economic disruption.

**Summary Points**

- There is growing recognition that primary prevention, including behavior change, must be central in the fight against HIV/AIDS. The earlier successes in Thailand and Uganda may not be fully relevant to the severely affected countries of southern Africa.
- We conducted an extensive multi-disciplinary synthesis of the available data on the causes of the remarkable HIV decline that has occurred in Zimbabwe (29% estimated adult prevalence in 1997 to 16% in 2007), in the context of severe social, political, and economic disruption.
- The behavioral changes associated with HIV reduction—mainly reductions in extramarital, commercial, and casual sexual relations, and associated reductions in partner concurrency—appear to have been stimulated primarily by increased awareness of AIDS deaths and secondarily by the country's economic deterioration. These changes were probably aided by prevention programs utilizing both mass media and church-based, workplace-based, and other inter-personal communication activities.
- Focusing on partner reduction, in addition to promoting condom use for casual sex and other evidence-based approaches, is crucial for developing more effective prevention programs, especially in regions with generalized HIV epidemics.

## Background

While dramatic gains in the availability of antiretroviral medications in developing countries have been achieved [1], there is growing consensus that, unless prevention efforts can be made more effective, there will ultimately be no victory in the fight against HIV/AIDS [1]–[4]. Maintaining tens of millions of people on treatment throughout their lifetimes will not be sustainable or affordable, particularly as drug resistance may increasingly result in the need for much more expensive second and third line medications. Although there have been promising breakthroughs in a few other areas, notably male circumcision and prevention of mother-to-child transmission (PMTCT) [1],[2],[5], it is widely recognized that behavior change must remain the core of prevention efforts [2]–[4].

While the often cited prevention success stories of Thailand [6] and Uganda [7],[8] are inspiring and informative, some of the specific socio-cultural, historical, and other factors in the southern African region—now the global epicenter of the HIV pandemic—are distinctive. In these “hyper-endemic” settings, where adult HIV prevalence ranges from 12% to 26% [1], HIV transmission is highly generalized, whereas Thailand's epidemic was much more concentrated. There, HIV transmission was driven mainly by brothel-based sex work—enabling the aggressive “100 percent condom” programs to be feasible, enforceable, and effective [6],[9]. The unprecedented HIV decline and associated behavior change in Uganda, mainly involving large reductions in multiple sexual partnerships [2],[7]–[10], occurred some 20 years ago and under rather different contextual and programmatic circumstances.

More recent examples of HIV prevalence reduction are emerging, including from Kenya, Haiti, the Dominican Republic, Malawi, and Ethiopia [1]–[3],[10]–[12]. Given the severe HIV epidemics that continue to plague parts of sub-Saharan Africa, there is an urgent need for studies identifying the proximate as well as underlying causes for these encouraging trends. In this paper, we review and summarize the principal findings of our comprehensive interdisciplinary analysis (commissioned by two United Nations agencies, the United Nations Populations Fund [UNFPA] and United Nations HIV-AIDS Program [UNAIDS]) of the causes behind the considerable HIV decline in Zimbabwe, including evidence for changes in patterns of sexual behavior and the contextual and possible programmatic reasons for these changes, which we have published in other peer-reviewed journals [13]–[15]. Here we also consider some policy implications of these findings.

## Synthesis of Data

### Data

**Figure 1:**
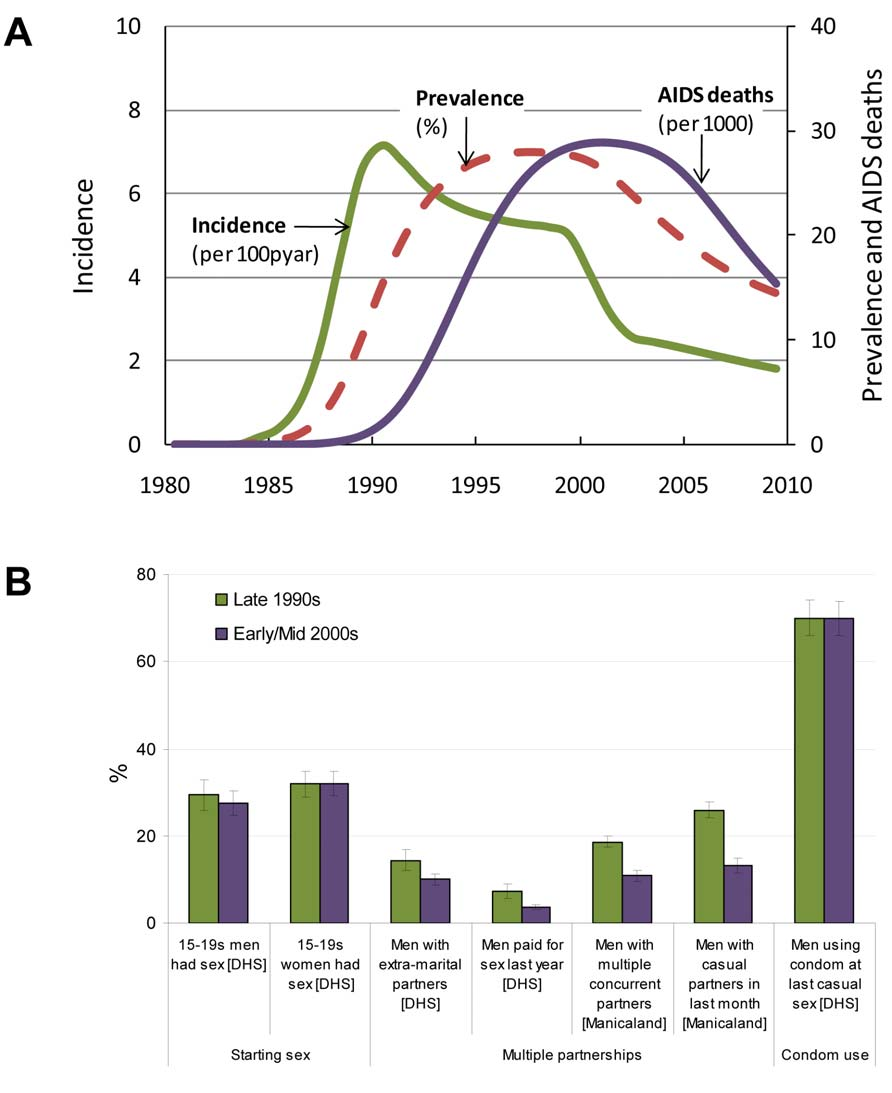
Summary of epidemiological findings. (A) Estimated trends in HIV prevalence, incidence, and AIDS deaths using a mathematical model of HIV transmission fitted to antenatal and household-based estimates of HIV prevalence, 1980–2010. HIV incidence peaks around 1991 and declines as part of the natural course of epidemic maturation; incidence decline is accelerated between about 1999 and 2003 due to reductions in sexual risk behavior [14]. (As has been noted [14], incidence declined a little earlier in urban areas. The model suggests behavior change could have continued partly into 2004 in rural areas, but the majority of changes were concentrated within the 1999–2003 period [14].) (B) Changes in key indicators of sexual partnership formation taken from the nationally representative DHSs (1999 and 2005/6) and surveys in Manicaland, rural eastern Zimbabwe (1998–2000 and 2001–2003) [13],[16].

**Figure 2:**
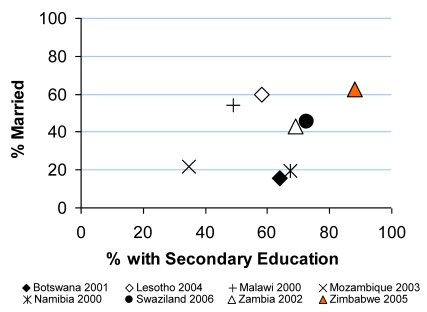
Levels of marriage and secondary education among men in urban areas in eight southern African countries. Estimates are for men aged 17–43 years (in Botswana, ages 14–48 years) in the years 2000–2006, chosen to maximize the overlap of temporal range between surveys and the age groups that contribute most to HIV transmission. All those with any secondary education were counted as having secondary education. “Married” category does not include those who were cohabiting but not married. Sources: DHS surveys performed in the years indicated in the legend, with the exception of Botswana (using the methodologically similar Botswana AIDS Impact Survey, 2001).

HIV prevalence data from national antenatal clinic surveillance and the household-based 2005/6 Demographic and Health Survey (DHS) were used to fit a mathematical model to estimate trends in HIV incidence and AIDS deaths in Zimbabwe (Figure 1A) [14]. Data from the DHS and other longitudinal surveys [13],[14],[16]–[18] were used to examine the possible contributions of changes in sexual behavior to reductions in HIV infection. Published data from focus group discussions with 90 adult men and 110 women in diverse urban and rural sites and several dozen in-depth key informant interviews as well as an extensive historical mapping of prevention programs [15], were examined in assessing the contributions of different contextual and programmatic factors to observed changes in behavior. Finally, DHS data on various potential proximal and contextual determinants of behavior change for Zimbabwe were compared with similar data for seven other southern African countries to identify distinctive patterns that might help to explain the earlier and faster HIV decline observed in Zimbabwe (Figures 2, S1).

### Process

Interpretation of data on the causes of HIV declines in other countries (e.g., Uganda) has proved to be contentious and problematic when drawing conclusions for policy. Therefore, to establish a consensus among key stakeholders on the roles of different potential causes of HIV decline in Zimbabwe, a national stakeholders meeting was held in Harare in May 2008 to examine the evidence assembled during this study. Stakeholders from a broad range of backgrounds attended the meeting, including high-level representatives of civil society and international organizations as well as senior non-political appointees within the Ministry of Health and Child Welfare and the National AIDS Council (participants and agenda listed in Text S2). At the meeting, the proximate and underlying contextual and programmatic factors that could have contributed to Zimbabwe's declining HIV epidemic were ranked systematically according to whether they were considered likely, plausible, or unlikely to be major contributors to the HIV decline, based upon triangulation of the data described above and from other relevant studies [13]–[19] (see Table 1 and Text S1). Four tests were applied in determining this ranking: 1) whether the factor could, based on the epidemiological literature, have been effective in reducing HIV risk at the population level; 2) for underlying factors, the existence of a clear causal pathway to those proximate factor(s) considered to be effective in reducing risk; 3) the extent or coverage of the factor; and 4) the consistency of timing between the primary change or amplification of the factor and the period of most significant risk reduction as estimated by the epidemiological model (about 1999–2003) [14]. Those factors considered effective that had already reached high levels of coverage before the period of most rapid risk reduction and continued to maintain high levels throughout this period were considered possible contributors to the HIV decline, as theoretically they could have helped to curb transmission (without an immediate impact on prevalence) so that later behavioral changes had a greater impact [20].

**Table 1:** Contributions of proximate causes to the HIV decline in Zimbabwe.

Proximate Cause	Population-Level Effectiveness^a^	Extent of Change^b^	Consistency in Timing of Change^c^	Major Contribution
**Behavioral**				
Age at first sex - postponement	Low	Low [QN]	Consistent	Unlikely
Partner numbers - reduction	High	High [QN & QL]	Consistent	Likely
Condom use - increase (in non-marital partnerships)	High (if consistent use)	Moderate [P, QN, QL]	Earlier	Plausible
**Biological**				
Transmission probability - reduction^d^	High	Low [QN & P]	Earlier	Unlikely

Form(s) of evidence supporting conclusion: M, epidemiological modeling; QN, survey data; P, program data; QL, qualitative data. See Text S1 for details.

a Extent to which changes in the factor concerned can reduce HIV transmission *at the population level*, as measured and modeled in scientific studies [M & QN].

b Extent to which changes in the given behavioral or biological determinant (by population sub-group) have occurred as observed in longitudinal surveys and/or program data.

c Extent to which the changes in risk behavior etc. occurred during the period of most rapid reduction in risk as determined by the epidemiological modeling assessment (i.e., about 1999–2003).

d Transmission probability could be affected by, for example, levels of blood safety, prevalence of other sexually transmitted infections, HIV medications, or male circumcision.

## Trends in HIV Prevalence and Incidence and Sexual Behavior in Zimbabwe

HIV prevalence in Zimbabwe increased rapidly in the early to mid-1990s, before reaching a plateau in the late 1990s (peaking at an estimated 29% adult prevalence in 1997 [13]), and declining after 2000 (down to 16% estimated prevalence in 2007). Mathematical modeling fitted to surveillance data [14] (Figure 1A) estimates that HIV incidence peaked around 1991 and (as in many other African countries [21]) declined gradually thereafter, mainly as part of the natural course of the epidemic, primarily due to saturation of infection in high-risk populations [11],[14],[22]. Between about 1999 and 2003, the pace of incidence decline accelerated considerably, which empirical data [13],[14] and modeling [14] suggest corresponded to reduced levels of risky sexual behavior.

As illustrated in Table 1 (and see Text S1), the unanimous conclusion from the stakeholders meeting (Text S2) held to assess, triangulate, and interpret the evidence assembled in the review [13]–[18] was that a reduction in multiple sexual partnerships was the most likely proximate cause for the recent decline in HIV risk. Although the DHS surveys indicate there was little change in age of sexual debut between 1999 and 2005/6, an approximately 30% reduction in the proportion of men reporting extra-marital partners occurred (Figure 1B). Similar or larger reductions in multiple partnerships among adults have been reported in other national surveys [13],[18]. Since presumably nearly all married people would have sex at least occasionally with their spouses (and given that most adults in Zimbabwe are married), this implies a sizeable reduction in the level of concurrent partnerships, which is a key epidemiological factor [23]. At a rural eastern Zimbabwean research site, where HIV prevalence fell substantially between 1998–2000 and 2001–2003, the fraction of men (and women; data not shown here) reporting concurrent partnerships declined by about 40% (Figure 1B) [16]. In addition, there were considerable reductions reported around this time in the number of Zimbabwean men paying for sex (Figure 1B) [13]. Participants in focus groups and key informant interviews conducted in various rural and urban areas similarly reported that major changes in norms of sexual behavior had occurred, especially after the late 1990s [15]. For example, many informants recounted that, whereas in earlier years it was commonplace for men gathering at locales such as beer halls to be surrounded by women/sex workers, by the late 1990s this norm had changed and it was now typical for men to gather strictly among themselves at such places. Moreover, while earlier it had been considered a proof of masculinity to acquire a sexually transmitted infection (STI), more recently getting an STI (and visiting sex workers) is typically said to be embarrassing or even shameful for Zimbabwean men [15].

## Reasons for HIV Decline and Associated Behavior Change

In fact, the prevalence of other STIs was greatly reduced during the early 1990s, mainly due to widespread syndromic management services [15]. Although STI control remains an important public health measure, the data from clinical trials regarding the population-level impact on HIV incidence are increasingly unconvincing (Text S1) [2],[24]. However, it has been hypothesized that STI treatment during the early phases of an HIV epidemic may help to reduce transmission (although this is unconfirmed by observational evidence; e.g., given the absence of HIV declines in several other African countries that had also implemented early and aggressive STI control programs). Reported condom use increased steadily during the 1990s (reaching 59% among men for last non-marital sexual encounter in the 1994 DHS), but did not increase further between 1999 and 2005/6 (Figure 1B), and remained very low for regular partnerships [13]. However, there is some evidence for modest improvement in the *consistency* of condom use among women in casual partnerships [13],[16], a more important measure for reducing infection risk than reported use at last sex [9].

In assessing the underlying factors for the national prevalence decline (Table 2, Figure S2), high AIDS mortality appears to have been the dominant factor for stimulating behavior change. Both empirical and modeling-derived estimates indicate that AIDS deaths increased dramatically during the mid-to-late 1990s, before stabilizing after 2000 [13],[14]. Moreover, men and women in focus groups and interviews repeatedly and consistently reported personal exposure to AIDS mortality and the resulting fear of contracting the virus to be the primary motivation for changes in sexual behavior, particularly reductions in casual sex and other multiple sexual partnerships [15].

**Table 2:** Contributions of underlying factors and programs to the HIV decline in Zimbabwe.

Underlying or Programmatic Cause	Causal Pathway	Population-Level Effectiveness^a^	Exposure/Coverage	Consistency in Timing of Change^b^	Major Contribution
**Mortality trends**					
AIDS deaths became much more noticeable	Close relatives & friends (& babies) dying, funerals → fear → behavior change [QL]	High [QL]	General population [M, QN, QL]	Consistent	Likely (primary)
**Socio-economic changes**					
Economic decline/increasing poverty	Less disposal income → ↓ commercial/extramarital sex [QL]	High [QL]	General population [QN, QL]	Consistent/later	Likely
**Behavior change programs**					
Mass media	Info/changes in social norms → behavior change [QL]	Potentially high [QL]	General population [QN, P, QL]	Gradual (early 1990s→)	Plausible^c^
Church teaching & programs	Info/changes in social norms → behavior change [QL]	Potentially high [QL]	General population [QN, P, QL]	Gradual (early 1990s→)	Plausible^c^
Workplace & other interpersonal communication	Info/changes in social norms → behavior change [QL]	Potentially high [QL]	General population [QN, P, QL]	Gradual, ↑ after late 1990s	Plausible^c^
School & other youth programs	Info/changes in social norms → behavior change [QL]	Potentially high [QL]	Youth [P, QL]	Earlier (early 1990s→)	Plausible^c^
Sex workers & clients (peer education, etc.)	↑Consistent condom use, ↓sex work visits	?	Urban core/bridge populations [P,QL]	Gradual	Plausible
Condom programming	↑Consistent condom use (for casual sex)	Moderate	Casual relationships [QN, P, QL]	Gradual (early 1990s→)	Plausible
Counseling and testing	↑Knowledge of HIV status → behavior change (in HIV+s)	Low	General population [QN, P, QL]	Scaled-up after 2002	Unlikely
**Biomedical interventions**					
Blood/injection safety	↓Transmission probability	High	Transfusion recipients [P]	Early (1980s→)	Unlikely
Treatment of sexually transmitted diseases	↓Transmission probability	Low?	STI patients [P, QL]	Early (late 1980s→)	Unlikely
Prevention of mother-to-child transmission	Fewer long-term survivors from infant infection	Low (in adults)	Infants [P]	Scaled-up after 2003	Unlikely
Antiretroviral medications	↓Transmission probability	Low	PLWHA [P]	Scaled-up after 2005	Unlikely

Form(s) of evidence supporting conclusion: M, epidemiological modeling; QN, survey or other quantitative data; P, program data; QL, qualitative data. See Text S1 for details.

a Extent to which change in the factor concerned is likely to effect behavior change, and thereby reduce HIV transmission at the population level.

b Extent to which the intervention was scaled-up during the period of most rapid reduction in risk as determined by the epidemiological modeling assessment (i.e., about 1999–2003).

c Behavior change programs as a whole probably contributed to reducing HIV risk but, given the limitations in the available data, it was not possible to isolate the contributions (if any) of each individual program area.

“?” indicates greater uncertainty.

The severe economic decline, taking hold in the late 1990s/early 2000s, appears to have played a considerable secondary role in amplifying patterns of behavior change, particularly partner reduction. Gross domestic product in Zimbabwe began to slump in the late 1990s, declining by about 40% between 1999 and 2005, with average real earnings plummeting by 90% during the same period. And many men in focus groups and interviews reported that having less disposable income has increasingly led to reduced ability to purchase sex or maintain multiple sexual relationships [15]. However, the most severe financial declines occurred after 2002, i.e., after the bulk of HIV incidence decline had evidently already occurred [13],[14] (Figures 1A, S3). The severe economic and political instability in the country also led to extensive international migration from Zimbabwe around this time. To have contributed substantially to the HIV decline, migration also would have needed to have been highly concentrated among individuals with HIV [13]. In fact, the available data suggest that the opposite was probably the case; e.g., HIV prevalence among pregnant Zimbabwean women living in the United Kingdom peaked at 12%, less than half the equivalent figure seen among pregnant women living in Zimbabwe itself [13]. Therefore, although the political and economic crises in Zimbabwe have been (especially in more recent years) extremely turbulent and of grave concern for humanitarian and other reasons, these factors do not appear to be the *predominant* ones for explaining the HIV decline that occurred.

In considering the potential impact of prevention programs on the HIV decline (Table 2, Figure S2), condom distribution and promotion efforts commencing in the early 1990s may have contributed through helping build high levels of condom use for commercial and casual sex [13],[15]. (And it appears that condoms were usually not promoted in the often highly “sexy” manner as occurred in some neighboring countries such as Botswana, but generally as a strictly “protective” public health intervention.) Voluntary counseling and testing (VCT) and PMTCT programs were, however, unlikely to have contributed significantly to HIV incidence decline as they were scaled-up only after 2002 [15]. Furthermore, the epidemiological evidence for individual- and population-level impact of VCT remains uncertain or weak [2],[25],[26]. During the 1990s, a wide range of prevention and information programs were implemented utilizing the national media along with school-, workplace-, and church-based activities, peer education, and other inter-personal communication interventions [15]. Community-based activities were intensified following establishment of the National AIDS Council in the late 1990s. This range of broader HIV education and prevention programming could have had impact. Focus group and interview participants mentioned a number of prevention programs and awareness/education efforts and many reported that the “B” part of “ABC” was promoted by churches in particular and was “heard” by many community members [15], yet no specific intervention was cited consistently.

## Why Has HIV Declined More in Zimbabwe than in Other Southern African Countries?

One question arising from this review is why similarly high AIDS mortality and extensive coverage of HIV prevention programs (resulting in similarly high levels of reported condom use, early and large reductions in STI incidence, etc.) in several other countries in the region have not yet led to substantial declines in HIV prevalence (or multiple sexual partnerships) [3],[7],[9],[21]. Our comparative analysis of eight southern African countries revealed few patterns of association. The HIV epidemic in Zimbabwe is somewhat older than in some other countries in the region, yet HIV prevalence has been declining markedly for over a decade now, which has not occurred to nearly the same extent, for example, in Malawi and Zambia (where HIV arrived even earlier). In addition to the severe economic decline, where Zimbabwe does stand out is in having high levels of both secondary education and marriage, especially in urban men, among whom the greatest level of behavior change evidently has occurred [13],[15],[19] (Figures 2, S1). It appears that this unique combination helped facilitate: 1) a clearer understanding and acceptance of how HIV is sexually transmitted (once such information became widely available through various AIDS education and prevention programs commencing in the early 1990s [15]), as some studies of schooling levels and HIV determinants have suggested [27] and 2) a greater ability to act upon “be faithful” messages, given the stronger marriage pattern [28]–[30] in Zimbabwe than that in neighboring countries also having relatively well-educated populations, such as Botswana and South Africa.

In addition, national survey data suggest that between the mid-1990s and the early 2000s, Zimbabweans increasingly received information about AIDS from their friends, churches, and other inter-personal (as compared to official media) sources (Figure S4) [15],[17]. A similar pattern has been linked to behavior change in Uganda [7],[31]. Furthermore, the Zimbabwean government's early adoption of a home-based care policy [32] may inadvertently have accelerated the process of behavior change. It has been hypothesized that, when people die at home, this direct confrontation with AIDS mortality is more likely to result in a tangible fear of death among family and friends than when patients are primarily cared for in clinical facilities, such as in Botswana [31].

It appears that the motivation for behavior change largely arose endogenously from within the population, and may have been partly due to events specific to Zimbabwe, such as the drastic economic decline in recent years. Nevertheless, it is unlikely that significant changes in behavior in response to the increasing levels of mortality could have occurred unless prevention programs had provided effective information and education about the link between risky sexual behavior and AIDS. We had hoped that our review would identify some particularly effective approaches, which could then be strengthened in Zimbabwe and inform prevention programs in other countries. Perhaps one reason that most respondents failed to identify specific effective programs is because it was the cumulative exposure to many programs that helped create a “tipping point” leading to changes in behavioral norms. We also note that government and civil society did promote faithfulness (mainly in the context of a generic “ABC” message), although not as early or as vigorously as Uganda's “zero grazing” campaign during the late 1980s [7],[8],[31]. Furthermore, findings from the qualitative research suggested the considerable impact of popular culture that occurred precisely around the key period of behavioral change of the late 1990s and early 2000s. For example, a (donor-sponsored) documentary *Todii (“What shall we do?”)* and a related widely popular song released by the famous performer Oliver Mutukudzi addressed the behavioral risks and social consequences of HIV infection [15].

## Implications for Improving HIV Prevention Efforts in Africa

The behavior changes associated with the HIV decline in Zimbabwe appear to have resulted primarily from increased interpersonal communication about HIV and its association with risky sexual behavior, due to high personal exposure to AIDS mortality and correct understanding of sexual HIV transmission (due to relatively high education levels along with information provided by HIV communication programs), as well as the deteriorating economic situation. However, the substantial shift in social norms that appears to have occurred, such as STI infection having become a cause for shame, suggests that the economic decline was probably more a co-facilitating factor rather than the major reason for behavior change; e.g., reduced income may prevent men from frequenting bars, but wouldn't change their attitude about having an STI.

One lesson emerging from this review is that in Zimbabwe, as elsewhere [2],[3],[7]–[10], partner reduction appears to have played a crucial role in reversing the HIV epidemic. Public and private sector programs in Zimbabwe are now building upon this knowledge by more assertively warning against multiple and concurrent partners and promoting sexual fidelity, in addition to consistent condom use and other effective approaches such as male circumcision [33]. Similar efforts have begun appearing elsewhere in the region, such as a bold Swaziland campaign highlighting the danger of having “secret lovers” [2],[3],[34].

A clear consensus was established regarding the conclusions presented in this article at the stakeholders' meeting, following extensive and open debate. Nevertheless, some uncertainty may remain regarding the conclusions reached. We hope that the detailed documentation of the data and criteria used in attributing causality provided here—and in the supporting publications, references, and supplementary text—will allow others to judge for themselves whether they agree with these conclusions.

HIV prevalence has declined in Zimbabwe by approximately 50%. This decline is almost unique in sub-Saharan Africa and it is hoped that the findings presented here may provide important insights for HIV control within the region. Additional investigations, similarly involving rigorous triangulation of data from multiple sources, should be commissioned in other countries where HIV prevalence has also declined substantially.

## Supporting Information

Figure S1 Levels of marriage and secondary education in eight southern African countries. Same as for Figure 2, except these data also include for (A) urban men; (B) urban women; (C) rural men; and (D) rural women.
(0.02 MB PDF)

Figure S2 Relationships between proximal and distal factors for behavior change and HIV decline in Zimbabwe. This chart illustrates the need to consider different levels of analysis, and suggests that at each level of analysis (including prevalence decline, incidence decline, behavior change, program activities, and the underlying socio-economic/cultural factors) a combination of a few key factors appears to best explain the observed changes.
(0.06 MB PDF)

Figure S3 Trends in economic indicators in Zimbabwe, 1990–2005 [35],[36]. Values shown in billions of Zimbabwean dollars at constant prices.
(0.01 MB PDF)

Figure S4 Sources of information on HIV-AIDS among young men in Zimbabwe. Sources: 1994 ZDHS; 2001/2 Zimbabwe Young Adult Survey [17]. Note that the categories included in the two surveys are not exactly identical. In the 2001/02 YAS “print media” and “hospital” were used (as per chart), but the 1994 DHS had asked for “newspaper” and “health worker,” respectively.
(256 KB PDF)

Text S1 Evidence and rationale for designations in Table 1 and Table 2.
(0.17 MB PDF)

Text S2 List of meeting participants and agenda from the May 2008 Stakeholders meeting, Harare.
(0.03 MB PDF)

## Abbreviations

DHS: Demographic and Health Survey
PMTCT: prevention of mother-to-child transmission
STI: sexually transmitted infection
VCT: voluntary counseling and testing

## References

[1] UNAIDS (the Joint United Nations Program on HIV/AIDS) (2010). AIDS epidemic update. UNAIDS publication UNAIDS/07.27E/JC1322E.

[2] Potts M, Halperin DT, Kirby D, Swidler A, Marseille E (2008). Reassessing HIV prevention.. Science.

[3] Southern African Development Community (SADC) (2006). Expert think tank meeting on HIV prevention in high-prevalence countries in Southern Africa: report. 10–12 May 2006; Maseru, Lesotho.

[4] Halperin DT, Steiner MJ, Cassell MM, Green EC, Hearst N (2004). The time has come for common ground on preventing sexual transmission of HIV.. Lancet.

[5] Nolan ML, Greenberg AE, Fowler MG (2002). A review of clinical trials to prevent mother-to-child HIV-1 transmission in Africa and inform rational intervention strategies.. AIDS.

[6] Nelson KE, Celetano DD, Eiumtrakol S, Hoover DR, Beyrer C (1996). Changes in sexual behaviors and a decline in HIV infection among young men in Thailand.. New Eng J Med.

[7] Stoneburner RL, Low-Beer D (2004). Population-level HIV declines and behavioral risk avoidance in Uganda.. Science.

[8] Green EC, Halperin DT, Nantulya V, Hogle JA (2006). Uganda's HIV prevention success: the role of sexual behavior change and the national response.. AIDS Behav.

[9] Hearst N, Chen S (2004). Condom promotion for AIDS prevention in the developing world: is it working?. Stud Fam Plann.

[10] Shelton JD, Halperin DT, Nantulya V, Potts M, Gayle HD (2004). Partner reduction is crucial for balanced ‘ABC’ approach to HIV prevention.. BMJ.

[11] Hallett TB, Aberle-Grasse J, Bello G, Boulos LM, Cayemittes MP (2006). Declines in HIV prevalence can be associated with changing sexual behavior in Uganda, urban Kenya, Zimbabwe, and urban Haiti.. Sex Transm Infect.

[12] Halperin DT, de Moya A, Perez-Then E, Pappas G, Garcia Calleja JM (2009). Understanding the HIV epidemic in the Dominican Republic: A prevention success story in the Caribbean?. J Acquir Immune Defic Syndr.

[13] Gregson S, Gonese E, Hallett TB, Taruberekera N, Hargrove JW (2010). HIV decline in Zimbabwe due to reductions in risky sex? Evidence from a comprehensive epidemiological review.. Internat J Epid.

[14] Hallett TB, Gregson S, Mugurungi O, Gonese E, Garnett GP (2009). Is there evidence for behavior change affecting the course of the HIV epidemic in Zimbabwe? A new mathematical modelling approach.. Epidemics.

[15] Muchini B, Benedikt C, Gregson S, Gomo E, Mate R (2010). Local perceptions of the forms, timing and causes of behavior change in response to the AIDS epidemic in Zimbabwe.. AIDS Behav.

[16] Gregson S, Garnett GP, Nyamukapa CA, Hallett TB, Lewis JJ (2006). HIV decline associated with behavior change in Eastern Zimbabwe.. Science.

[17] Zimbabwe Ministry of Health and Child Welfare and Centers for Disease Control and Prevention (2004). Young adult survey 2001–2002.

[18] Population Services International (2003). Knowledge, attitudes, beliefs and practices on HIV/AIDS in Zimbabwe.

[19] Lopman B, Lewis J, Nyamukapa C, Mushati P, Chandiwana S, Gregson S (2007). HIV incidence and poverty in Manicaland, Zimbabwe: is HIV becoming a disease of the poor?. AIDS.

[20] Garnett GP, Anderson RM (1995). Strategies for limiting the spread of HIV in developing countries: conclusions based on studies of the transmission dynamics of the virus.. J Acquir Immune Defic Syndr Hum Retrovirol.

[21] Shelton JD, Halperin DT, Wilson D (2006). Has global HIV incidence peaked?. Lancet.

[22] Anderson RM, Medley GF, May RM, Johnson AM (1986). A preliminary study of the transmission dynamics of the human immunodeficiency virus (HIV), the causative agent of AIDS.. IMA J Math Appl Med Biol.

[23] Mah TL, Halperin DT (2010). Concurrent sexual partnerships and the HIV epidemics in Africa: Evidence to move forward.. AIDS Behav.

[24] Gray RH, Wawer JM (2008). Reassessing the hypothesis on STI control for HIV prevention.. Lancet.

[25] Corbett EL, Makamure B, Cheung YB, Dauya E, Matambo R (2007). HIV incidence during a cluster-randomized trial of two strategies providing voluntary counseling and testing at the workplace, Zimbabwe.. AIDS.

[26] Shelton J (2008). Counselling and testing for HIV prevention.. Lancet.

[27] Hargreaves JR, Bonell CP, Boler T, Boccia D, Birdthistle I (2008). Systematic review exploring time trends in the association between educational attainment and risk of HIV infection in sub-Saharan Africa.. AIDS.

[28] Spark-du Preez N, Zaba B, Nyamukapa CA, Mlilo M, Gregson S (2004). “Kusvika taparadzaniswa nerufu” (Until death do us part).. Afr J AIDS Res.

[29] Bongaarts J (2007). Late marriage and the HIV epidemic in sub-Saharan Africa.. Popul Stud.

[30] Carael M, Ali M, Cleland J (2001). Nuptiality and risk behaviour in Lusaka and Kampala.. Afr J Reprod Health.

[31] Low-Beer D, Stoneburner R, Poku NK, Whiteside A (2004). Uganda and the challenge of AIDS.. Political economy of AIDS in Africa. Chapter 10.

[32] Hansen K, Woelk G, Jackson H, Kerkhoven R, Manjonjori N (1998). The cost of home-based care for HIV/AIDS patients in Zimbabwe.. AIDS Care.

[33] Ministry of Health and Child Welfare (2008). Zimbabwe national HIV prevention strategy document.

[34] Spina A (2009). Secret lovers kill: a national mass media campaign to address multiple and concurrent partnerships.. AIDSTAR-One: Case Studies Series.

[35] United Nations Development Programme (UNDP) (2008). Comprehensive economic recovery in Zimbabwe. UNDP report [see Figure 2.8].

[36] International Monetary Fund (IMF) (2008). World economic outlook database, October 2008.

