# Supplementary material for: A Surprising Prevention Success: Why Did the HIV Epidemic Decline in Zimbabwe?
Source: PLoS Med. 2011 Feb 8;8(2):e1000414. doi: 10.1371/journal.pmed.1000414 (PMC3035617; doi:10.1371/journal.pmed.1000414)
Supplement: Figure S1 — Levels of marriage and secondary education in eight southern African countries. Same as for Figure 2, except these data also include for (A) urban men; (B) urban women; (C) rural men; and (D) rural women. (0.02 MB PDF) [file pmed.1000414.s001.pdf]

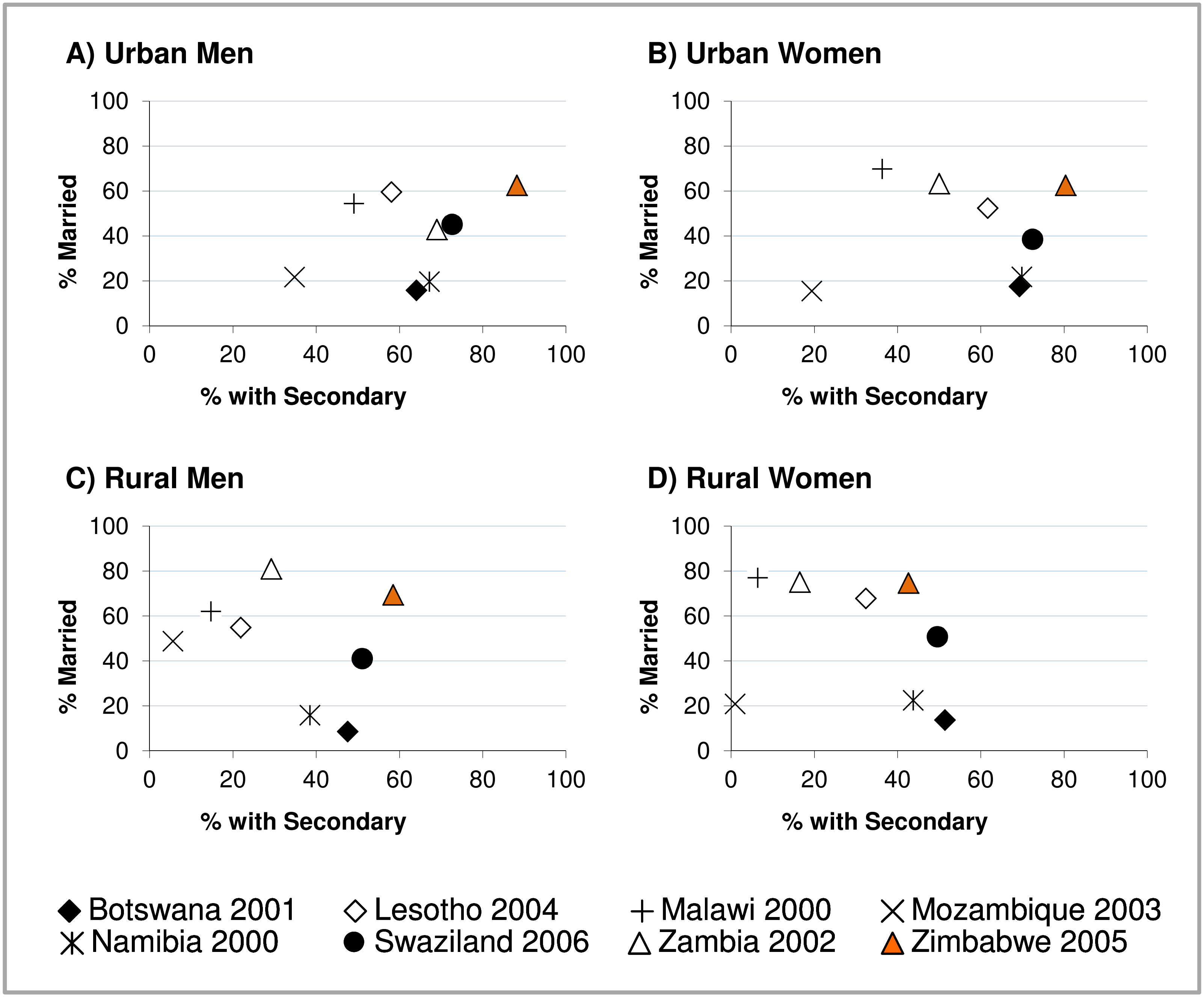
A) Urban Men
B) Urban Women
C) Rural Men
D) Rural Women
% Married
% with Secondary
0
20
40
60
80
100
Botswana 2001
Lesotho 2004
Malawi 2000
Mozambique 2003
Namibia 2000
Swaziland 2006
Zambia 2002
Zimbabwe 2005
