# Supplementary material for: A Surprising Prevention Success: Why Did the HIV Epidemic Decline in Zimbabwe?
Source: PLoS Med. 2011 Feb 8;8(2):e1000414. doi: 10.1371/journal.pmed.1000414 (PMC3035617; doi:10.1371/journal.pmed.1000414)

Figure S2: Relationships between Proximal and Distal Factors

For Behavior Change and HIV Decline in Zimbabwe

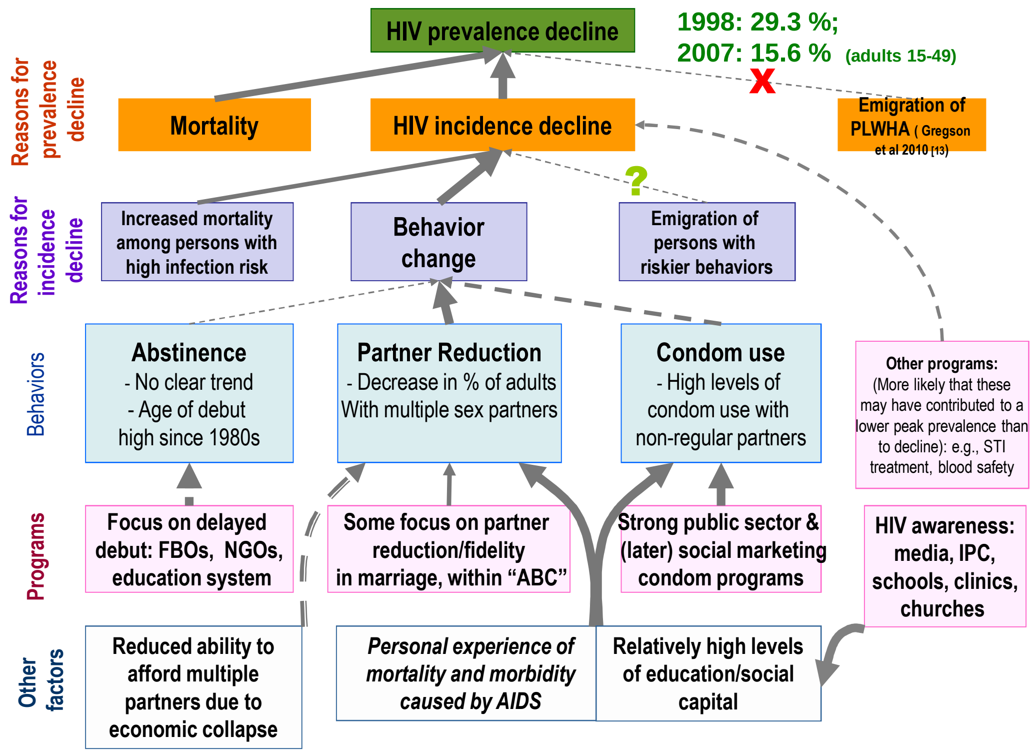

Supplement: Figure S2 — Relationships between proximal and distal factors for behavior change and HIV decline in Zimbabwe. This chart illustrates the need to consider different levels of analysis, and suggests that at each level of analysis (including prevalence decline, incidence decline, behavior change, program activities, and the underlying socio-economic/cultural factors) a combination of a few key factors appears to best explain the observed changes. (0.06 MB PDF) [file pmed.1000414.s002.pdf]
