# Supplementary figures and images for: A Surprising Prevention Success: Why Did the HIV Epidemic Decline in Zimbabwe?
Source: PLoS Med. 2011 Feb 8;8(2):e1000414. doi: 10.1371/journal.pmed.1000414 (PMC3035617; doi:10.1371/journal.pmed.1000414)

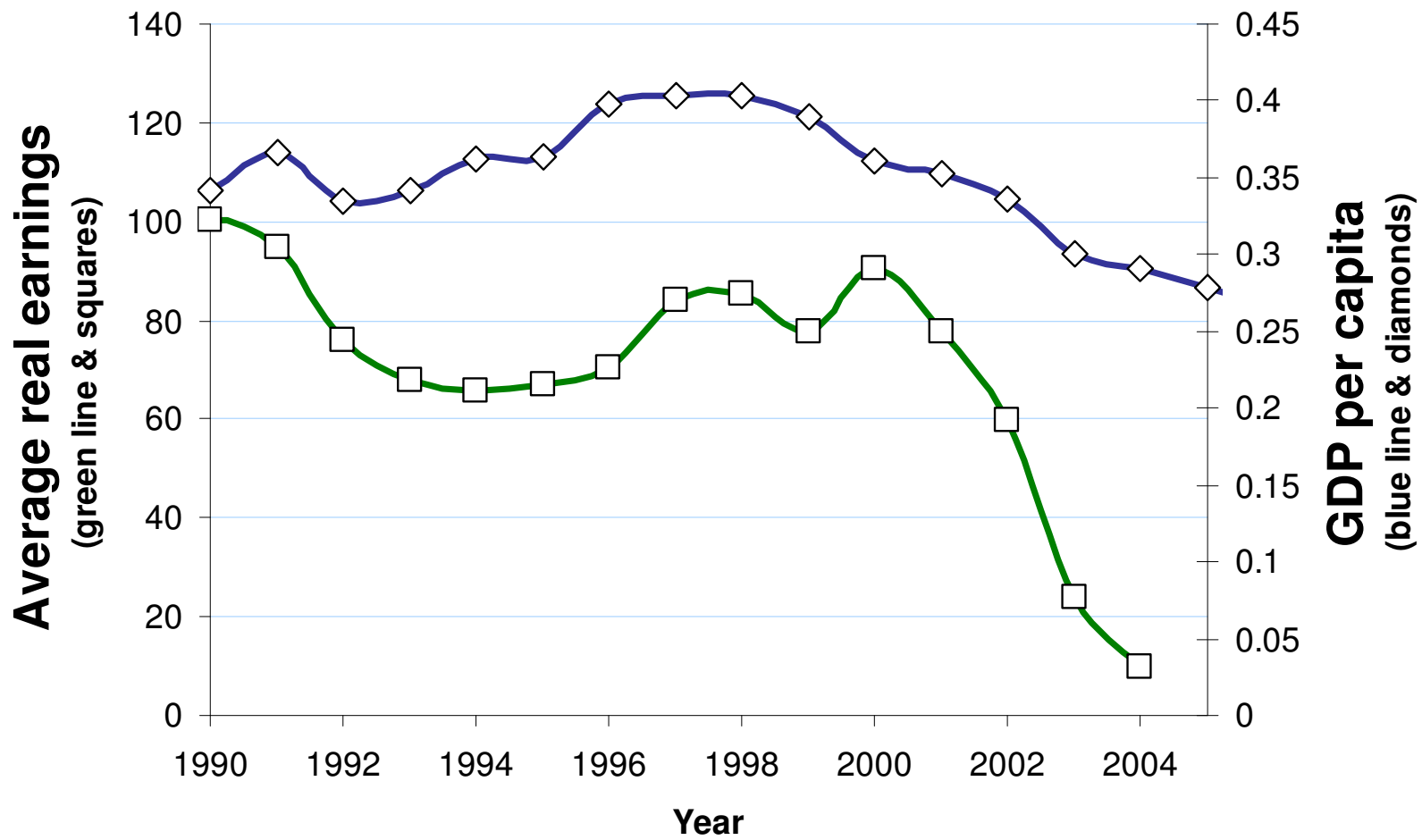

Supplement: Figure S3 — Trends in economic indicators in Zimbabwe, 1990–2005 [35],[36]. Values shown in billions of Zimbabwean dollars at constant prices. (0.01 MB PDF) [file pmed.1000414.s003.pdf]

**Percentage of men 15-29 who ever received HIV/AIDS information from selected sources**

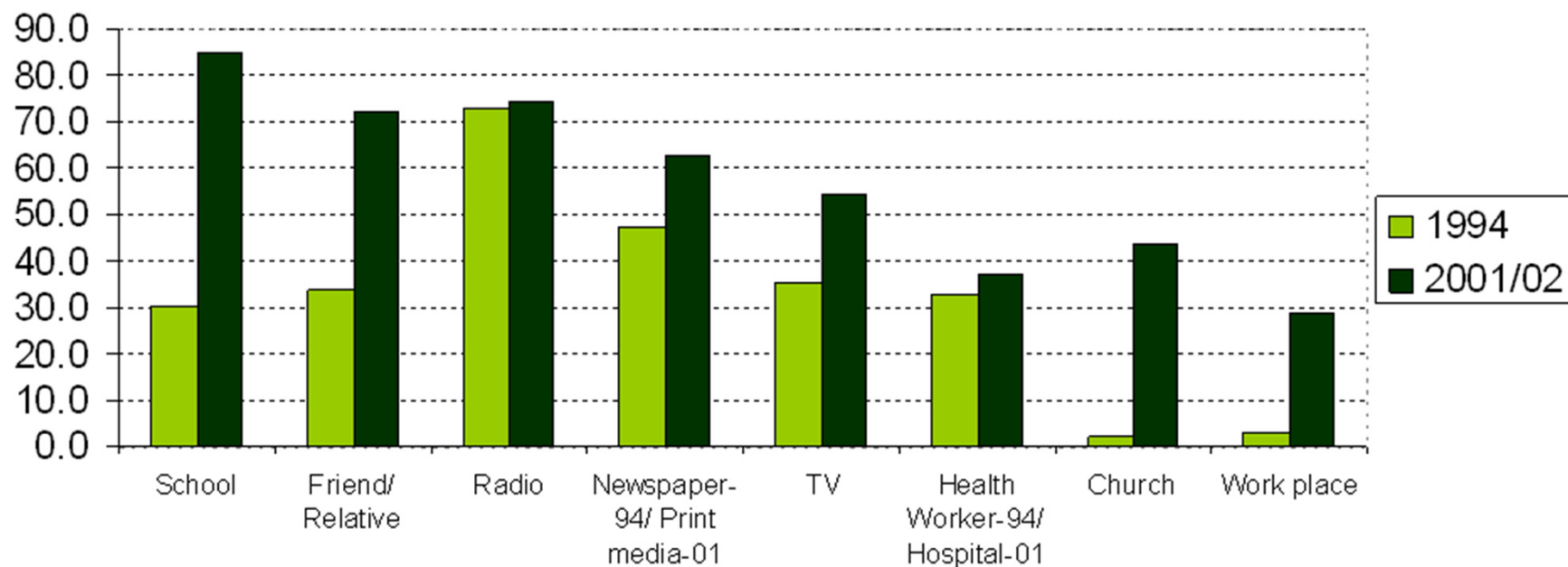

Supplement: Figure S4 — Sources of information on HIV-AIDS among young men in Zimbabwe. Sources: 1994 ZDHS; 2001/2 Zimbabwe Young Adult Survey [17]. Note that the categories included in the two surveys are not exactly identical. In the 2001/02 YAS “print media” and “hospital” were used (as per chart), but the 1994 DHS had asked for “newspaper” and “health worker,” respectively. (256 KB PDF) [file pmed.1000414.s004.pdf]
