# Supplementary material for: A Surprising Prevention Success: Why Did the HIV Epidemic Decline in Zimbabwe?
Source: PLoS Med. 2011 Feb 8;8(2):e1000414. doi: 10.1371/journal.pmed.1000414 (PMC3035617; doi:10.1371/journal.pmed.1000414)
Supplement: Text S1 — Evidence and rationale for designations in Table 1 and Table 2. (0.17 MB PDF) [file pmed.1000414.s005.pdf]

TEXT S1 for:

A Surprising Prevention Success?: Why Did the HIV Epidemic Decline in Zimbabwe

Evidence and Rationale for Designations in Tables 1 and 2:

Table 1:

Age at first sex – postponement

Population level-effectiveness: Low. At the individual level, abstinence or postponement of age at first sex is associated with reductions in HIV infection in youth [Supplemental Text ref. #S1, S2]. However, modeling and empirical studies indicate that lifetime risk of infection and population-level transmission are not substantially reduced by later sexual debut [S3-S5].

Extent of change: Low. No change in reported age at first sex between Zimbabwe Demographic and Health Surveys (ZDHS) in 1999 and 2005/6 (Figure 1B in the main text). Modest delay in age at first sex among men in rural Manicaland study [Main Text ref. #T16].

Consistency of timing of change: Consistent. Change in Manicaland recorded between late 1990s and early 2000s [T16].

Major contribution: Unlikely.

Multiple sexual partnerships – reduction

Population level-effectiveness: High. Primary determinant of individuals' risk of HIV infection and of the rate at which infections spread through populations, including the importance of concurrent partnerships [S6-S10]. Reductions in multiple partnerships have been associated with declines in HIV prevalence in other settings [T2,T6-T8,T21,S10].

Extent of change: High. Substantial reductions in all indicators of multiple partnerships in ZDHS and Manicaland surveys (see Figure 1A in main text) and other sources [T5,T18]. Frequently cited in qualitative research (key informant interviews and focus-group discussions) [T15].

Consistency of timing of change: Consistent. Change in ZHDS and Manicaland surveys and qualitative research reported between late 1990s and early-to-mid 2000s.

Major contribution: Likely.

Condom use – increase (in casual partnerships)

Population level-effectiveness: High (for consistent use). Reviews of empirical data [T12,S11,S12] and modeling studies [S13] indicate that consistent condom use results in significant reduction in HIV transmission.

Extent of change: Moderate. Condom use at last casual sex was high in 1999 but did not increase further by 2005/6 (Figure 1B in main text). Relatively high levels of condom use were already recorded in the 1994 ZDHS (59% for men at last non-marital sexual encounter). The consistency with which condoms are used did increase modestly in the Manicaland study between 1998-2000 and 2001-2003 [T16]. Increase in condom use was occasionally cited in focus-group discussions [T15].

Consistency of timing of change: Earlier. Reported condom use increased most rapidly before 1998, the approximate start of the period of largest reduction in risk (T14).

Major contribution: Plausible. Condom use may have contributed to the HIV decline through: (i), curbing transmission in the mid 1990s (even without an immediate effect on prevalence) so that later behavioral changes may have had a greater impact [T20]; or, (ii) possible increase in consistency of condom use after 1998 [T16].

Transmission probability - reduction

This includes factors that can modify the probability that HIV is transmitted at each sexual contact (i.e., the prevalence of other sexually transmitted infections [STIs], use of HIV medications, and male circumcision) or procedures to ensure blood safety (i.e. screening for HIV in blood transfusions, and precautions to prevent contaminated medical injections).

Population level-effectiveness: High. Transmissibility of HIV is a key determinant of the extent of infectious spread [T22,S7,S8] and most of the factors listed above can substantially modify the probability of HIV transmission. [S14-19].

Extent of change: Low. Reductions in the prevalence of Chancroid and other STIs did occur in the early 1990s [T5,T15], but there is conflicting evidence about whether this would translate into a substantial reduction in the overall probability of HIV transmission [T2,T24,S17-19]. The prevalence of male circumcision is unlikely to have changed substantially in Zimbabwe (circumcision prevalence was only ~10% in the 2005/6 ZDHS). The availability of antiretroviral therapy was very low in Zimbabwe before 2005 [T15,S20]. Improvements in blood safety would only directly lead to reduced risk of infection among blood transfusion recipients, meaning that the impact across the greater population would also have been small, and this mainly occurred back in the 1980s [T15]. Contaminated medical injections could have had greater potential for a substantial impact [S21] and currently account for only a small number of infections [S22] but, again, intensified preventative precautions were already instituted back in the mid-1980s [T15].

Consistency of timing of change: Early. Screening of blood donations and measures to prevent contaminated medical injections commenced in the mid-1980s.

Major contribution: Unlikely.

Table 2

Mortality Trends

AIDS deaths became much more noticeable

Causal pathway: Close relatives, friends and babies dying, leading to common experience of suffering and attending funerals, and consequently a great fear of becoming infected [S23].

Population-level effectiveness: Frequently cited in interviews and focus-group discussions as a major factor in prompting changes in behavior [T15]. Some quantitative evidence for an impact on HIV incidence amongst closely affected individuals in Zimbabwe was found in one rural study [S24]. Associations between exposure to AIDS deaths and behavior change have also been identified in other African settings [T7,T11,S25].

Exposure: Empirical data [T5] and modeling projections [T14] indicate that the AIDS mortality rate increased dramatically in the 1990s, and in surveys and focus-group discussions it was frequently reported that many people in the general population personally knew someone who had died of AIDS [T15].

Consistency in timing of change: AIDS deaths peaked sometime between the late 1990s and early 2000s [T5], consistent with the estimated period of most rapid reduction in risk behavior [T14]. In the qualitative research, AIDS mortality was cited as the main factor prompting behavior change, especially since the late 1990s [T15].

Major contribution: Likely (primary).

Socioeconomic Changes

Economic decline/increasing poverty

Causal pathway: Less disposal income, leading to reduced ability to pay for commercial sex or support extramarital partnerships.

Population-level effectiveness: High. Multiple sexual partnerships have been found to be closely associated with prevalent and incident HIV infection [S6-9], and transactional sex/ability to pay for sex is commonly associated with higher numbers of sex partners [S26].

Exposure: Frequently cited in qualitative research [T15], and the scale of economic decline (see Figure S1) suggests that many people in the general population who had been able to support extramarital and other casual and commercial partnerships became unable to do so.

Consistency in timing of change: Consistent/later. Both per-capita Gross Domestic Product and average real earnings plummeted in the period after the late 1990s, though the most severe declines occurred mainly after about 2002 (Figure S1).

Major contribution: Likely.

Behavior Change Programs

Mass media/Church teaching & programs/Workplace & other interpersonal-based programs

Causal pathway: Behavior change prompted directly via information and prevention messages received, or indirectly through broader changes in social norms [T7,T11].

Population-level effectiveness: Potentially high. Greater awareness about modes of HIV transmission and participation in community–based programs can help reduce risk of infection at the individual and population level [T7,S6,S25]. However, trials of interpersonal-based interventions in Africa have thus far failed to show an impact at the population level [S27,S28]. Few data on the impact of exposure to specific programs in Zimbabwe are available.

Exposure: There was widespread access to mass media and some other education and prevention approaches in the general population (Figure S3) [T15].

Consistency in timing of change: Gradual increase in mass media and other general population campaigns beginning in the early 1990s. These programs preceding the period of most rapid risk reduction but could potentially have helped lay the groundwork for later reductions in risky behavior. Also, between 1994 and 2001/2 there was a large increase in the proportion of young men who reported receiving HIV/AIDS information from churches (Figure S3). Workplace-based and some other interpersonal interventions were scaled-up gradually from the late 1990s, in part reflecting the establishment of the National AIDS Council, the National AIDS Trust Fund and other community-based activities [T15].

Major contribution: Plausible.

School and youth programs

Causal pathway: Behavior change prompted directly through information received, or indirectly through changes in social norms [T7,T11].

Population-level effectiveness: Potentially high. Higher levels of school education may be starting to become associated with lower risk of HIV infection in Africa [T27], and greater access to education combined with AIDS clubs and other community participation was associated with improved knowledge, reduced risk behavior and lower risk of having acquired HIV infection in young women in a study in eastern Zimbabwe [S29]. The only published randomized trial of an adolescent sexual health intervention found some impact on reported behavior but not on HIV or STI incidence [S30]. No data on the impact of the national school-based HIV-AIDS programs in Zimbabwe are available.

Exposure: Youth.

Consistency in timing of change: Gradual. School-based programs were scaled-up in the early-mid 1990s [T15]. Cohorts of school-age children in the early/mid 1990s became young adults during the period of most rapid risk decline (late 1990s/early 2000s).

Major contribution: Plausible – in combination with other behavior change programs.

Sex workers & clients (peer education, etc.)

Causal pathway: Intensive promotion of consistent condom use, also resulting in reduced numbers of sex worker visits.

Population-level effectiveness: Uncertain. Sex work is thought to play a central role in HIV transmission in concentrated epidemics and probably in early-stage generalized epidemics, but less so in mature generalized epidemics [S31]. In the rural Manicaland region, a peer-education intervention among sex workers aimed at reducing the frequency of unprotected sex did not reduce HIV incidence in the wider community [S27]. However, in another region of Zimbabwe, it was estimated that 20% of prevalent HIV infections among men could be attributed to sex work [S32].

Exposure: Urban core/bridge populations. In general, sex work is expected to be more common in urban than in rural areas. Bridge populations comprise men who have unprotected intercourse with sex workers and also with their regular partners as well as with casual partners [S33].

Consistency in timing of change: Gradual increase, beginning in the early 1990s. This precedes the period of most rapid risk reduction, but earlier changes could potentially have enhanced the impact of later reductions in HIV risk.

Major contribution: Uncertain.

Condom programming

Causal pathway: Messages promoting condom use (especially for non-regular partnerships) and increased availability in high-risk settings leading to greater and more consistent utilization.

Population-level effectiveness: Moderate. Increases in consistent condom use can substantially reduce HIV transmission [T12,S11-13], especially in concentrated epidemics, where most HIV transmission results from commercial or casual sex. However, it is unclear whether sufficiently high levels of consistent condom use have been achieved in any generalized epidemic (where much transmission occurs within more regular -- and typically concurrent – partnerships) to have a significant impact at the population level [T2,T3,T12]. (See other details regarding condom use under Table 1.)

Exposure: Mainly those in commercial and other casual partnerships.

Consistency in timing of change: Gradual increase, beginning in the early 1990s. Sales of social marketed condoms increased rapidly only after 2003 [T15]

Major contribution: Plausible.

HIV counseling and testing

Causal pathway: More people knowing their infection status and receiving counseling, leading to changes in risk behavior (especially among those found to be HIV-positive).

Population-level effectiveness: Low. Behavioral changes following voluntary counseling and testing (VCT) in Zimbabwe have been modest (or possibly even negative) [T25, S34], as elsewhere in Africa [T26], and mathematical models suggest that such levels of change would not to lead to substantially reduced transmission of HIV [S35].

Exposure: General population, although disproportionately among the seriously ill.

Consistency in timing of change: VCT services were only scaled-up in Zimbabwe after 2002 [T15], following the period of most rapid risk reduction.

Major contribution: Unlikely.

Biomedical Interventions

Blood/injection safety

Causal pathway: Reduction in HIV transmission during blood transfusion or medical injection [S36].

Population-level effectiveness: High. Measures that were taken were likely to have removed the risk of HIV contamination in blood and injection equipment [S37].

Exposure: Those receiving transfusions and medical injections.

Consistency in timing of change: Programs started from the mid-1980s [T15], well before the period of most rapid risk reduction.

Major contribution: Unlikely.

Treatment of other sexually transmitted infections

Causal pathway: Reduction in prevalence of sexually transmitted infections that enhance transmissibility of or susceptibility to HIV, leading to an overall reduction in the probability of HIV transmission.

Population-level effectiveness: Uncertain. (See details regarding other STIs in the “transmission probability” section under Table 1.)

Exposure: STI patients in the general population.

Consistency in timing of change: Programs started from the late 1980s [T15], before the period of most rapid risk reduction.

Major contribution: Unlikely.

Prevention of mother-to-child transmission

Causal pathway: More long-term survivors from infant HIV infection. Reductions in subsequent risk behavior in pregnant women receiving VCT.

Population-level effectiveness: Low (in adults). Single-dose Nevirapine (NVP) for mother and baby is used in the national program. This regimen had an efficacy of 41% in a clinical trial in Uganda [S38]. However, the population-level impact of NVP-based programs is low in the absence of high coverage and adherence [S39]. Furthermore, estimates of long-term survivorship following peri-natal infection are low [S40]. Scientific studies suggest limited efficacy of VCT in reducing adult risk behavior (see previous section on HIV counseling and testing).

Exposure: Infants

Consistency in timing of change: Prevention of mother-to-child transmission programs were scaled-up only after 2003 [T15].

Major contribution: Unlikely.

Antiretroviral medications

Causal pathway: Reductions in viral load resulting in a lower probability of HIV transmission from individuals previously at more advanced stages of infection/disease.

Population-level effectiveness: Low. Mathematical modeling suggests that antiretroviral treatment, commenced when or after symptoms of immune-suppression develop, will have a limited impact on the overall population-level transmission of HIV [S41].

Exposure: People living with AIDS.

Consistency in timing of change: Programs were scaled up only after 2005 [T15], substantially after the period of most rapid risk reduction.

Major contribution: Unlikely.

References

1. Pettifor AE, van der Straten A, Dunbar MS, Shiboski SC, Padian NS. **Early age at first sex: a risk factor for HIV infection among women in Zimbabwe**. *AIDS* 2004,18:1435-1442.
2. Hallett TB, Lewis JJ, Lopman BA, Nyamukapa CA, Mushati P, Wambe M, *et al.* **Age at first sex and HIV infection in rural Zimbabwe**. *Stud Fam Plann* 2007,38:1-10.
3. Hallett TB, Gregson S, Lewis JJC, Lopman BA, Garnett GP. **Behaviour change in generalised HIV epidemics: impact of reducing cross-generational sex and delaying age at sexual debut**. *Sex Transm Infect* 2007,83:i50-54.
4. Gregson S, Nyamukapa CA, Garnett GP, Mason PR, Zhuwau T, Carael M, *et al.* **Sexual mixing patterns and sex-differentials in teenage exposure to HIV infection in rural Zimbabwe**. *Lancet* 2002,359:1896-1903.
5. Garnett GP, Anderson RM. **Factors controlling the spread of HIV in heterosexual communities in developing countries: patterns of mixing between different age and sexual activity classes**. *Philos Trans R Soc Lond B Biol Sci* 1993,342:137-159.
6. Lopman B, Nyamukapa CA, Mushati P, Wambe M, Mupambireyi Z, Mason PR, *et al.* **Determinants of HIV incidence after 3 years follow-up in a cohort recruited between 1998 and 2000 in Manicaland, Zimbabwe**. *International Journal of Epidemiology* 2008,37:88-105.
7. Quigley M, Munguti K, Grosskurth H, Todd J, Mosha F, Senkoro K, *et al.* Sexual behaviour patterns and other risk factors for HIV infection in rural Tanzania: a case-control study. *AIDS* 1997,11:237-248.
8. Morris M, Kretzschmar M. **Concurrent partnerships and the spread of HIV**. *AIDS* 1997,11:641-648.
9. Bassett MT, McFarland WC, Ray S, Mbizvo S, Machekano R, van de Wijgert JHHM, Katzenstein DA. **Risk factors for HIV infection at enrolment in an urban male factory cohort in Harare, Zimbabwe**. *Journal of Acquired Immune Deficiency Syndromes and Human Retrovirology* 1996,13:287-293.
10. Halperin DT, Epstein H. **Why is HIV prevalence so severe in southern Africa? The role of multiple concurrent partnerships and lack of male circumcision: Implications for AIDS prevention.** *South African J HIV Med* 2007;26**:**19–25; www.harvardaidsprp.org/research/halperin&epsteinwhy-is-hiv-prevalence-so-severe.pdf.
11. Holmes KK, Levine R, Weaver M. **Effectiveness of condoms in preventing sexually transmitted infections**. *Bull World Health Organ* 2004,82:454-461.
12. Pinkerton SD, Abramson PR. **Effectiveness of condoms in preventing HIV transmission**. *Soc Sci Med* 1997,44:1303-1312.
13. Bracher M, Santow G, Watkins SC. **Assessing the potential of condom use to prevent the spread of HIV: a microsimulation study**. *Stud Fam Plann* 2004,35:48-64.
14. Baggaley RF, Boily M-C, White RG, Alary M. Risk of HIV-1 transmission for parenteral exposure and blood transfusion: a systematic review and meta-analysis. *AIDS* 2006,20:805-812.
15. Auvert B, Taljaard D, Lagarde E, Sobngwi-Tambekou J, Sitta R, Puren A. **Randomized, controlled intervention trial of male circumcision for reduction of**

HIV infection risk: the ANRS 1265 trial. *Public Library of Science Medicine* 2005,2:e298.

16. Weiss HA, Halperin D, Bailey RC, Hayes R, Schmid G, Hankins CA. **Male circumcision for HIV prevention: from evidence to action?** *AIDS* 2008,22:567–574.
17. Latif AS, Katzenstein DA, Bassett MT, Houston S, Emmanuel JC, Marowa E. **Genital ulcers and transmission of HIV among couples in Zimbabwe**. *AIDS* 1989,3:519-523.
18. Cameron DW, Simonsen JN, D'Costa LJ, Ronald AR, Maitha GM, Gakinya MN, *et al.* **Female to male transmission of HIV-1: risk factors for seroconversion in men**. *The Lancet* 1989,2(8660):403-407.
19. Rottingen JA, Cameron DW, Garnett GP. **A systematic review of the epidemiologic interactions between classic sexually transmitted diseases and HIV: how much really is known?** *Sex Transm Dis* 2001,28:579-597.
20. WHO, UNAIDS, UNICEF. **Towards Universal Access: Scaling up priority HIV/AIDS interventions in the health sector Progress Report (available from http://www.who.int/hiv/mediacentre/univeral_access_progress_report_en.pdf)**. UN, Geneva; April, 2007.
21. White RG, Cooper BS, Kedhar A, Orroth KK, Biraro S, Baggaley RF, *et al.* **Quantifying HIV-1 transmission due to contaminated injections**. *Proceedings of the National Academy of Sciences* 2007,104:9794-9799.
22. Lopman B, Garnett GP, Mason PR, Gregson S. **Evidence of absence: injection history and HIV incidence in rural Zimbabwe**. *Public Library of Science* 2005,2:142-146.
23. McKusick L, Wiley JA, Coates TJ, Stall R, Saika G, Morin S, *et al.* **Reported changes in the sexual behavior of men at risk for AIDS, San Francisco, 1982-84--the AIDS Behavioral Research Project**. *Public Health Rep* 1985,100:622-629.
24. Lopman B, Nyamukapa CA, Mushati P, *et al.* **Determinants of HIV incidence after 3 years follow-up in a cohort recruited between 1998 and 2000 in Manicaland, Zimbabwe.** *Internat J Epidem* 2008,**37**:88-105.
25. Epstein H. *The invisible cure: Africa, the West and the fight against AIDS*. New York: Farrar Straus and Giroux, 2007.
26. Leclerc-Madlala S. **Transactional sex and the pursuit of modernity.** *Social Dynamics* 2003;29:1-21.
27. Kamali A, Quigley M, Nakiyingi JS, Kinsman J, Kengeya-Kayondo J, Gopal R, *et al.* **Syndromic management of STIs and behaviour change interventions on transmission of HIV-1 in rural Uganda: a community randomised trial.** *The Lancet* 2003,361:645-652.
28. Gregson S, Adamson S, Papaya S, Mundondo J, Nyamukapa CA, Mason PR, *et al.* **Impact and Process Evaluation of Integrated Community and Clinic-Based HIV-1 Control: A Cluster-Randomised Trial in Eastern Zimbabwe**. *PLoS Med* 2007,4:e102.
29. Gregson S, Terceira N, Mushati P, Nyamukapa CA, Campbell C. **Community group participation: can it help young women to avoid HIV? An exploratory study of social capital and school education in rural Zimbabwe.** *Social Science and Medicine* 2004,58:2119-2132.
30. Ross DA, Changalucha J, Obasi A, Todd J, Plummer ML, Cleophas-Mazige B, *et al.* **Biological and behavioural impact of an adolescent sexual health intervention in Tanzania: a community-randomized trial** *AIDS* 2007,21:1943-1956.
31. Boily MC, Lowndes C, Alary M. **The impact of HIV epidemic phases on the effectiveness of core group interventions: insights from mathematical models**. *Sex Transm Infect* 2002,78 Suppl 1:i78-90.

32. Cowan FM, Langhaug LF, Hargrove JW, Jaffar S, Mhuriyengwe L, Swarthout TD, *et al.* **Is sexual contact with sex workers important in driving the HIV epidemic among men in rural Zimbabwe?** *J Acquir Immune Defic Syndr* 2005,40:371-376.
33. Morris M, Podhisita C, Wawer MJ, Handcock MS. **Bridge populations in the spread of HIV/AIDS in Thailand**. *AIDS* 1996,10:1265-1271.
34. Sherr L, Lopman B, Kakowa M, Dube S, Chawira G, Nyamukapa C, *et al.* **Voluntary counselling and testing: uptake, impact on sexual behaviour, and HIV incidence in a rural Zimbabwean cohort**. *AIDS* 2007,21:851-860.
35. Hallett TB, Dube S, Cremin Í, *et al.* **The role of testing and counselling for HIV prevention and care in the era of scaling-up antiretroviral therapy**. *Epidemics*, in press.
36. Baggaley RF, Boily M-C, White RG, Alary M. **Risk of HIV-1 transmission for parenteral exposure and blood transfusion: a systematic review and meta-analysis**. *AIDS* 2006,20:805-812.
37. Lopman B, Garnett GP, Mason PR, Gregson S. **Evidence of absence: injection history and HIV incidence in rural Zimbabwe**. *Public Library of Science* 2005,2:142-146.
38. Jackson JB, Musoke P, Fleming T, et al. **Intrapartum and neonatal singledose nevirapine compared with zidovudine for prevention of mother-tochild transmission of HIV-1 in Kampala, Uganda: 18-month follow-up of the HIVNET 012 randomised trial**. *Lancet.* 2003,362:859-868.
39. Dube S, Boily M-C, Mugurungi O, Mahomva A, Chikhata F, Gregson S. **Estimating vertically acquired HIV infections and the impact of the prevention of mother-to-child transmission program in Zimbabwe: insights from decision analysis models.** *Journal of Acquired Immune Deficiency Syndromes* 2008,48:72-81.
40. Marston M, Zaba B, Salomon JA, Brahmbhatt H, Bagenda D. **Estimating the net effect of HIV on child mortality in African populations affected by generalized HIV epidemics**. *J Acquir Immune Defic Syndr* 2005,38:219-227.
41. Salomon JA, Hogan DR. **Evaluating the impact of antiretroviral therapy on HIV transmission**. *AIDS* 2008,22 Suppl 1:S149-159.
