# Supplementary material for: A Surprising Prevention Success: Why Did the HIV Epidemic Decline in Zimbabwe?
Source: PLoS Med. 2011 Feb 8;8(2):e1000414. doi: 10.1371/journal.pmed.1000414 (PMC3035617; doi:10.1371/journal.pmed.1000414)
Supplement: Text S2 — List of meeting participants and agenda from the May 2008 Stakeholders meeting, Harare. (0.03 MB PDF) [file pmed.1000414.s006.pdf]

Why has HIV prevalence declined in Zimbabwe?

Stakeholder Consultation on Findings from Epidemiological Modeling and Qualitative Research

Harare, 30 May 2008, Crown Plaza Hotel

Programme

Time	Agenda item	Chair
8.15	Registration	
8.30	Welcome and introduction (Chair)	Dr. O. Mugurungi
8.45	Objectives of the meeting, Dr. K. Ampomah	
9.00	Summary of findings of the 2005 epi-review; Dr. S. Gregson, BRTI	
9.30	Overview of HIV decline study phase 2, Dr. C. Benedikt, UNFPA	
9.40	Results from epidemiological modeling: HIV prevalence trends, Dr. T. Hallett, Imperial College	
10.10	Discussion on epidemiological trends – Consensus-building on key findings	
10.30	Tea	
10.45	Behavioural trends in survey and other study data, Dr. T. Hallett	Dr. K. Ampomah
11.15	Questions	
11.30	Qualitative research findings on trends in behaviours; Dr. D. Halperin; B. Muchini	
11.45	Discussion on behavioural trends - Consensus-building on key findings	
12.15	Qualitative research findings on underlying social, economic and cultural factors; Dr. D. Halperin, Harvard University/ B. Muchini, Consultant	
12.30	Discussion on trends in underlying factors - Consensus-building on key findings	
12.45	Lunch	
13.45	Historical mapping of interventions, Dr. C. Benedikt /B. Muchini	Dr. T Magure
14.15	Qualitative research findings on programme impact, B. Muchini	
14.30	Discussion on programme impact - Consensus-building on key findings	
15.00	Why Zimbabwe? Comparison of regional trends, Dr. D. Halperin, Harvard University	
15.30	Discussion	
15.45	Tea	
16.00	Conclusions: Dr. Simon Gregson	Dr. B. Campbell
16.30	Next steps: Dr. B. Campbell	
16.55	Closing remarks: Dr. Magure	
17.00	Closure	

Participants' List

Name	Organisation

1. Daniel Gapare	Batsirai Group
2. Jeremiah Chikovore	BRTI
3. Dr. Mamadou Diallo	CDC
4. Backson Muchini	Consultant
5. Nyasha Mayanga	DFID
6. Dr Daniel Halperin	Harvard University
7. Dr. Simon Gregson	Imperial College, London
8. Dr Timothy Hallett	Imperial College, London
9. Ticharwa Masimira	MASO
10. Sinokuthemba Xaba	MOHCW
11. Dr Owen Mugurungi	MOHCW
12. Elizabeth Gonese	MOHCW
13. Shungu Munyati	MRCZ
14. Oscar Mundida	NAC
15. Raymond Yekeye	NAC
16. Tendayi Westerhof	PPAAT
17. Karin Hatzold	PSI
18. Noah Taruberekera	PSI
19. Michael Chommie	PSI
20. Kumbirai Chatora	PSI
21. Lois Chingandu	SAfAIDS
22. Vivienne Kernohan	SAfAIDS
23. Emmanuel Baingana	UNAIDS
24. Hege Wågan	UNAIDS
25. Dr. Kwame Ampomah	UNAIDS
26. Dr Bruce Campbell	UNFPA
27. Dr Clemens Benedikt	UNFPA
28. Caroline Nyamayemombe	UNFPA
29. Sunday Manyenya	UNFPA
30. Helen Jackson	UNFPA-CST
31. Thembi Ziyambi	UNICEF
32. Lovemore Magwere	UNICEF
33. Prof Francis Onyango	WHO
34. Dr Christine Chakanyuka	WHO
35. Lindiwe Chaza-Jangira	ZAN
36. Francisca Binza	ZAN
37. Sherla Greenland	ZiCHIRe
38. Dr Stella Chiruva	ZNFPC
